# Facile synthesis of folate-conjugated magnetic/fluorescent bifunctional microspheres

**DOI:** 10.1186/1556-276X-9-558

**Published:** 2014-10-08

**Authors:** Mao Shen, Wenping Jia, Caiping Lin, Guodong Fan, Yangmin Jin, Xiaoying Chen, Guang Chen

**Affiliations:** 1 College of Pharmaceutical and Chemical Engineering, Taizhou University, Jiaojiang 318000, People’s Republic of China; 2 Key Laboratory of Auxiliary Chemistry and Technology for Chemical Industry, Ministry of Education, Shaanxi University of Science and Technology, Xi’an 710021, China; 3 College of Pharmaceutical Sciences Zhejiang University, Hangzhou 310058, China

**Keywords:** Magnetic nanoparticles, Chitosan, Solvothermal, Fluorescent

## Abstract

In this paper, we investigated the functional imaging properties of magnetic microspheres composed of magnetic core and CdTe quantum dots in the silica shell functionalized with folic acid (FA). The preparation procedure included the preparation of chitosan-coated Fe_3_O_4_ nanoparticles (CS-coated Fe_3_O_4_ NPs) prepared by a one-pot solvothermal method, the reaction between carboxylic and amino groups under activation of NHS and EDC in order to obtain the CdTe-CS-coated Fe_3_O_4_ NPs, and finally the growth of SiO_2_ shell vent the photoluminescence (PL) quenching via a Stöber method (Fe_3_O_4_-CdTe@SiO_2_). Moreover, in order to have a specific targeting capacity, the magnetic and fluorescent bifunctional microspheres were synthesized by bonding of SiO_2_ shell with FA molecules via amide reaction (Fe_3_O_4_-CdTe@SiO_2_-FA). The morphology, size, chemical components, and magnetic property of as-prepared composite nanoparticles were characterized by ultraviolet-visible spectroscopy, fluorescent spectroscopy, Fourier transform infrared spectroscopy (FTIR), X-ray powder diffraction (XRD), scanning transmission electron microscopy (SEM), transmission electron microscopy (TEM), thermogravimetric analysis (TGA), and vibrating sample magnetometer (VSM), respectively. The results show that the magnetic and fluorescent bifunctional microspheres have strong luminescent which will be employed for immuno-labeling and fluorescent imaging of HeLa cells.

## Background

In the past few years, a variety of ferroferric oxide magnetic nanoparticles (Fe_3_O_4_ NPs) have been widely used in biomedical applications, such as targeted drug delivery, rapid biological separation, biosensors, and magnetic hyperthermia therapy [1-4]. At the same time, quantum dots (QDs) have been actively studied for bioimaging applications due to their excellent optical properties such as narrow emission bands, continuous broad absorption band, and high resistance to photobleaching in comparison with organic dyes. Highly luminescent QDs could serve as luminescent markers, while Fe_3_O_4_ NPs could be easily manipulated under the external magnetic field. Therefore, combining QDs and Fe_3_O_4_ NPs to get fluorescent/magnetic bifunctional composite nanoparticles has attracted intense attention due to its appealing applications [5-7]. However, the preparation of fluorescent/magnetic bifunctional composite nanoparticles is challenging. There are also problems associated with their low chemical stability and biocompatibility. A specific difficulty in the preparation of fluorescent/magnetic bifunctional composite nanoparticles is the risk of quenching of the fluorophore on the particle surface by the magnetic core and the complicated synthesis procedures. These problems can be solved by coating the magnetic core with a stable isolating shell prior to the introduction of luminescent QDs (Fe_3_O_4_@X@QDs, X = SiO_2_[8], carbon [5], polymers [9], et al.). However, most of these conversion processes are very complicated and could cause dramatic changes on the size, shapes, and phases of the NPs, which are not conducive to biological applications. Thus, it is desirable to find a simple and flexible synthesis method to prepare the stable isolating shell.

In addition, folic acid is a small-molecule vitamin that is essential for the human body, especially the single carbon metabolism of eukaryotic cells and nucleoside synthesis. Folate receptor (FR) is a well-known tumor marker [10]; it has limited expression in normal tissues, and is greatly overexpressed in a variety of carcinomas. Folic acid (FA) conjugated of imaging agents have high binding affinity for cell surface FRs, which allows for selective targeting of tumor cells.

**Figure 1:**
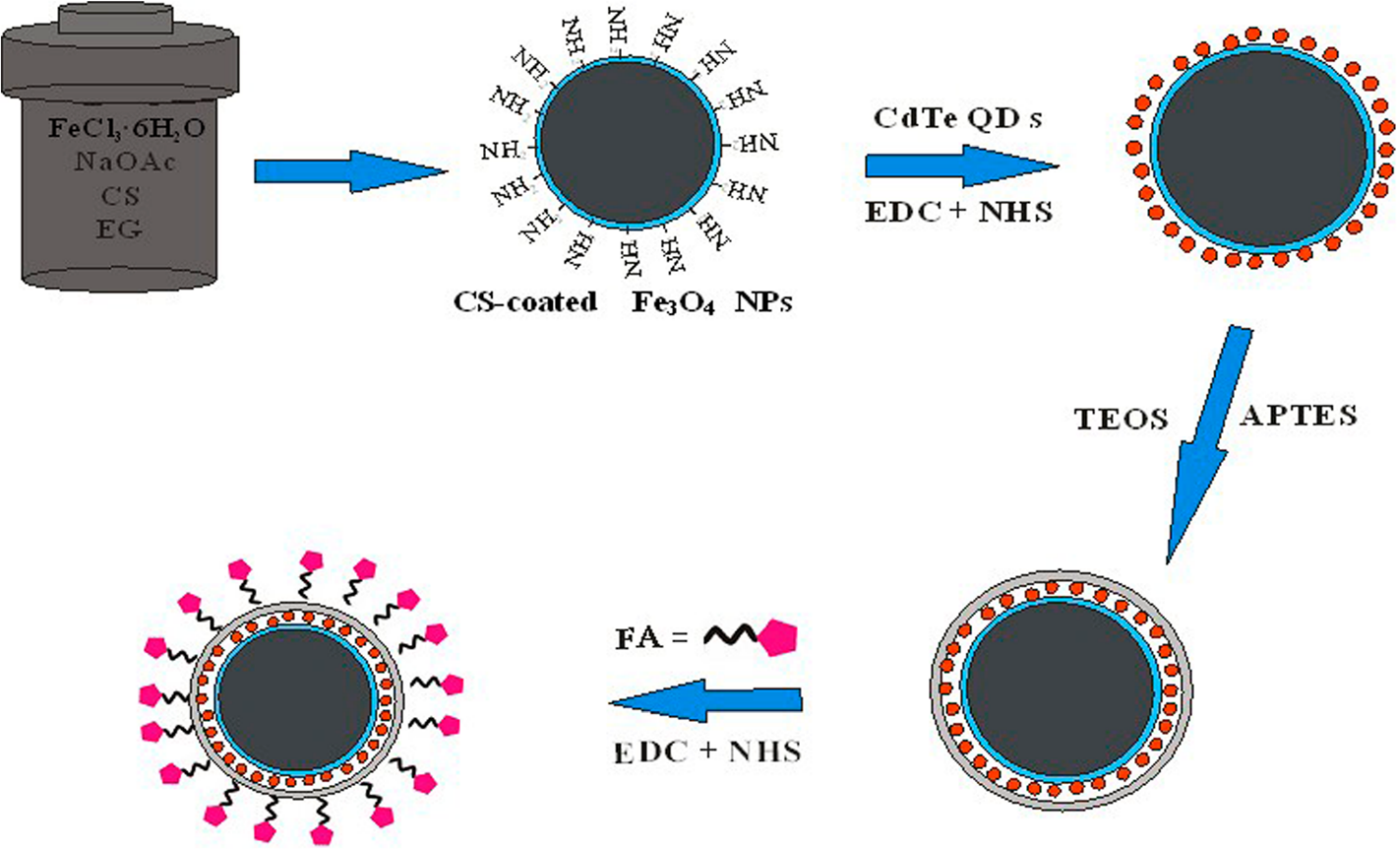
**The preparation procedure of the Fe**_**3**_**O**_**4**_**-CdTe@SiO**_**2**_**-FA microspheres.**

In this work, we report the synthesis and characterization of Fe_3_O_4_-CdTe@SiO_2_-FA microspheres. The preparation procedure of the Fe_3_O_4_-CdTe@SiO_2_-FA microspheres is shown in Figure 1.

## Methods

### Chemicals

Ferric chloride hexahydrate (FeCl_3_ · 6H_2_O, >99%), anhydrous sodium acetate (NaOAc), ethylene glycol (EG), cadmium chloride (CdCl_2_ · 2.5H_2_O), tellurium dioxide (TeO_2_, 99.99%), 3-mercaptopropionic acid (MPA, 99%), glucose, polyvinylpyrrolidone (PVP), FA, *N*-hydroxysuccinimide (NHS), and *N*-ethyl-*N*-(3-(dimethylamino) propyl) carbodiimide (EDC) were purchased from Aldrich (Wyoming, IL, USA). HeLa cells were supplied by Zhejiang University. Sodium hydroxide (NaOH) and aqueous ammonia solution (25 wt %) were analytical grade. The pure water was obtained from a Milli-Q synthesis system (Millipore, Billerica, MA, USA).

### Characterization methods

Fluorescence spectra were obtained at room temperature using a CARY ECLIPSE (Agilent Technologies, Santa Clara, CA, USA) fluorescence spectrometer. Transmission electron microscopy (TEM) images were obtained on a JEM-2100 TEM (Jeol Ltd., Tokyo, Japan). X-ray powder diffraction (XRD) analysis was performed using a Dmax-2500 (CuKα =1.5406 Å; Rigaku Corporation, Tokyo, Japan). Magnetic characteristics were studied using a vibrating sample magnetometer (VSM) (Lake Shore Company, Westerville, OH, USA) at room temperature. Scanning transmission electron microscopy (SEM) was carried out on a Philips XL30 microscope (Philips, Amsterdam, The Netherlands). The zeta potential of these particles was measured by dynamic light scattering (DLS) with a Delsa™ NanoC Particle Size Analyzer (Beckman Coulter, Pasadena, CA, USA). Thermogravimetric analysis (TGA) of nanocomposite and chitosan was performed on the TGA Q500 from TA Instruments (New Castle, DE, USA). Analyzed samples were heated from 100 to 800°C at a heating rate of 10°C/min under a nitrogen flow of 50 ml/min. The Fourier transform infrared spectroscopy (FTIR) of as-prepared composite nanoparticles was performed by Nicolet 5700 (Thermo Nicolet, Waltham, MA, USA).

### Synthesis of CS-coated Fe_3_O_4_ NPs

Functionalized magnetite nanoparticles were synthesized via a versatile solvothermal reaction as previously reported with a slight modification [11]. Typically, FeCl_3_ · 6H_2_O (1.50 g), chitosan (0.5 g), NaOAc (3.6 g), and PVP (1.0 g) were added to 70 ml of ethylene glycol to give a transparent solution via vigorous stirring. This mixture was then transferred to a Teflon-lined autoclave (80 ml) for treatment at 200°C for 8 h. The products were obtained with the help of a magnet and were exhaustively washed, in order for all the chitosan in the final products to be chemically bound to the magnetic nanoparticles. Finally, the products were collected with a magnet and dried in a vacuum oven at 60°C for further use.

### Synthesis of MPA-stabilized CdTe QDs

MPA-stabilized CdTe QDs were synthesized by the reaction of tellurium dioxide as a tellurium source and 3-mercaptopropionic acid as a reductant following our reported procedures [12]. Briefly, 2 mmol CdCl_2_ · 2.5H_2_O was dissolved in 100 ml of deionized water in a beaker, and 5.4 mmol MPA was added under stirring. The pH of the solution was then adjusted to 10.0 by dropwise addition of 0.1 mol/l NaOH solution. Under stirring, 0.5 mmol TeO_2_ was added to the original solution. The typical molar ratio of Cd^2+^/Te^2-^/MPA was 1:0.25:2.7. The monomer was heated in a XO-SM100 microwave-assisted heating system (XO-SM100 Microwave and Ultrasonic combination response system, MW-50%; Xianou Company, Nanjing, China). The solution was refluxed for 3.5 h and the reaction was terminated to obtain CdTe QDs with red-emitting colors.

### Synthesis of CdTe-CS-coated Fe_3_O_4_ NPs

The CdTe-CS-coated Fe_3_O_4_ microspheres were synthesized by the reaction between carboxylic and amino groups under activation of NHS and EDC. Briefly, 0.1 g chitosan-coated (CS-coated) Fe_3_O_4_ microspheres were dispersed in 100 ml deionized water, mixed with 0.1 g NHS, and 0.158 g EDC by ultrasonication for 30 min, then different amounts of previously obtained CdTe NCs solutions were added to the suspension under continuous mechanical stirring for 24 h. Finally, the products were collected with a magnet and dried in a vacuum oven at 50°C for further use.

### Synthesis of Fe_3_O_4_-CdTe@SiO_2_-NH_2_ core/shell microspheres

The Stöber method was employed to coat the as-produced spherical particles with a SiO_2_ layer. In this process, 0.1 g of CdTe-CS-coated Fe_3_O_4_ powder was dispersed in a mixture of ethanol (80 ml) and deionized water (20 ml) by ultrasonication for 10 min. Then, 1 ml of ammonia solution (25%) and 0.1 ml of TEOS were added into the solution quickly under continuous mechanical stirring. After 6 h of stirring at room temperature, 0.1 ml of 3-aminopropyltriethoxysilane (APTES) was added into the mixture solution and refluxed for 12 h. At last, the Fe_3_O_4_-CdTe@SiO_2_-NH_2_ microspheres were separated by a magnet and repeatedly washed with ethanol for several times and dried in a vacuum oven at 50°C for further use.

### Conjugation of Fe_3_O_4_-CdTe@SiO_2_-NH_2_ magnetic/fluorescent microspheres with FA

In order to test the applications of Fe_3_O_4_-CdTe@SiO_2_-NH_2_ microspheres in immuno-labeling and fluorescent imaging of cancer cells, the microspheres were conjugated with FA. The conjugation of FA with Fe_3_O_4_-CdTe@SiO_2_-NH_2_ microspheres was completed through the reaction between carboxylic and amino groups under activation of NHS and EDC. Typically, 50 mg Fe_3_O_4_-CdTe@SiO_2_-NH_2_ microspheres was dissolved in 100 ml dimethylsulphoxide (DMSO), mixed with 0.1 g FA, 0.05 g NHS, and 0.06 g EDC by ultrasonication for 30 min, then stirred overnight. The mixed solution was separated with an external magnetic field, alternately rinsed with ultra-pure water and ethanol four times, then dispersed in 50 ml deionized water for further use.

## Results and discussion

### Structure characterization of the nanoparticles

**Figure 2:**
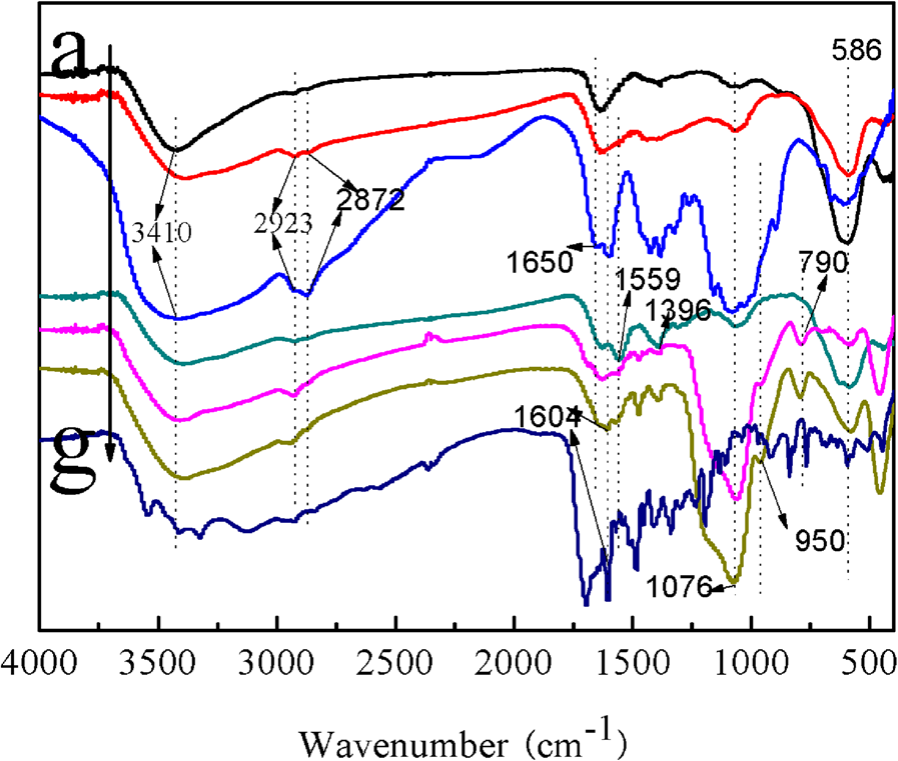
**The FI-IR spectra of (a) Fe**_**3**_**O**_**4**_**, (b) CS-coated Fe**_**3**_**O**_**4**_**, (c) CS, (d) CdTe-CS-coated Fe**_**3**_**O**_**4**_, **(e) Fe**_**3**_**O**_**4**_**-CdTe@SiO**_**2**_**-NH**_**2**_**, (f), Fe**_**3**_**O**_**4**_**-CdTe@SiO**_**2**_**-FA, and (g) FA.**

The FTIR spectra of naked Fe_3_O_4_, CS, FA, CS-coated Fe_3_O_4_, CdTe-CS-coated Fe_3_O_4_ NPs, Fe_3_O_4_-CdTe@SiO_2_-NH_2_, and Fe_3_O_4_-CdTe@SiO_2_-FA are given in Figure 2. In the spectrum of naked Fe_3_O_4_ (Figure 2a), the characteristic Fe-O group band of naked Fe_3_O_4_ appeared at 590 cm^-1^[11]. For pure CS (Figure 2f), it can be seen that there is a broad band at 3,410 cm^-1^ assigned to the O-H stretching vibration, and the C-H group is manifested through peaks 2,923 and 2,872 cm^-1^. Of the characteristic absorption peaks of the primary amine (-NH_2_), one overlaps with the -OH band at 3,410 cm^-1^, and the second is visible at 1,650 cm^-1^. Compared with the naked Fe_3_O_4_ NPs, the band around 1,072 cm^-1^ is the stretching vibrations of the C-O bond which is stronger in Figure 2b, suggesting the existence of C-O-C group in CS [13]. The FTIR spectra of CdTe-CS-coated Fe_3_O_4_ NPs was showed in Figure 2d, where the bands at the peaks at 1,559 and 1,396 cm^-1^ corresponding to the stretching vibration of carboxyl salt are found, which indicates the presence of carboxyl groups on the surface of the CdTe-CS-coated Fe_3_O_4_ NPs composite nanoparticles. The FTIR spectrum of Fe_3_O_4_-CdTe@SiO_2_-NH_2_ (Figure 2e) shows the main characteristic band of the symmetric and asymmetric stretching vibration of framework and terminal Si-O-groups at 790, 950, and 1,076 cm^-1^. As shown in Figure 2c, after conjugation with FA, the spectrum of Fe_3_O_4_-CdTe@SiO_2_-FA NPs exhibits the characteristic absorption peaks of FA at 1,604 cm^-1^, in which the absorption bands is assigned to the conjugated double absorption of benzene.

**Figure 3:**
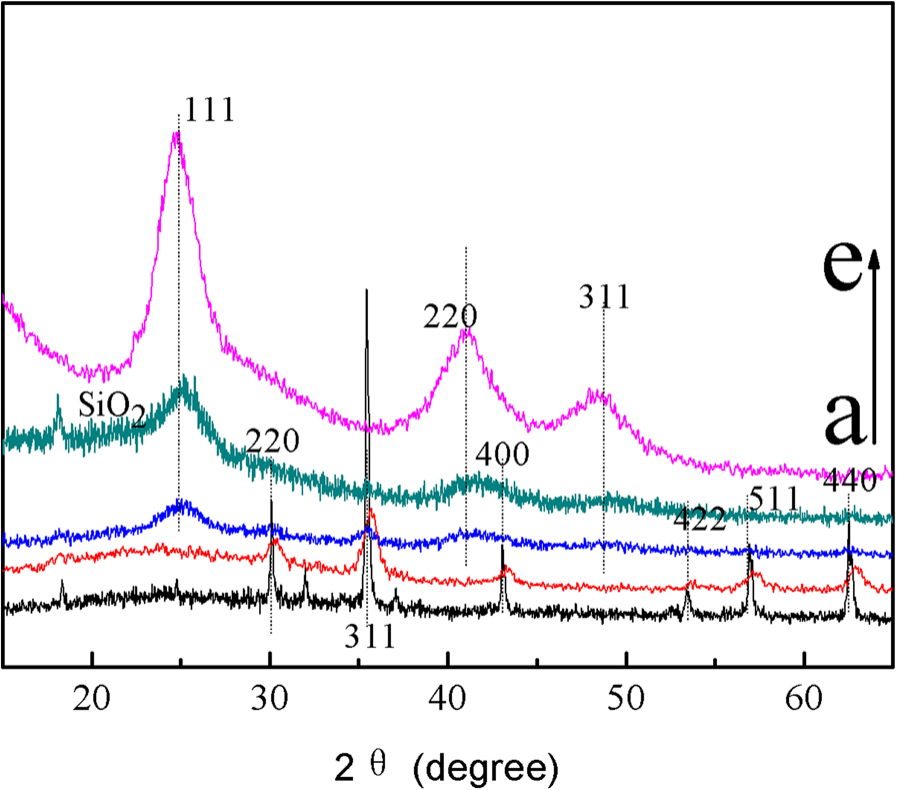
**XRD patterns of the synthesized: (a) naked Fe**_**3**_**O**_**4**_**, (b) CS-coated Fe**_**3**_**O**_**4**_**, (c) CdTe-CS-coated Fe**_**3**_**O**_**4**_**, (d) Fe**_**3**_**O**_**4**_**-CdTe@SiO**_**2**_**-NH**_**2, **_**and (e) CdTe QDs.**

The crystal structures of the composite magnetic nanoparticles were characterized by X-ray diffraction in Figure 3. For naked Fe_3_O_4_ NPs as prepared in this work, the six characteristic peaks for 2*θ* =30.08°, 35.42°, 43.08°, 53.56°, 56.98°, and 62.62° marked by their indices (220), (311), (400), (422), (511), and (440) were observed [14]. As shown in Figure 3b,c, these characteristic peaks can be seen in the composite magnetic nanoparticles. The XRD pattern of CdTe is shown in Figure 3e. The diffraction peaks at 2*θ* =24.535°, 40.589°, and 48.037° can be readily assigned to the (111), (220), and (311) planes, respectively. The position of the XRD peaks matches well those of the bulk CdTe cubic structure (JCPDS no. 19-0629). As shown in Figure 3c,d, these characteristic peaks can be seen in the composite magnetic nanoparticles. In addition, a broad peak around 2*θ* =20 to 25° corresponding to the amorphous phase of silica (JCPDS card 01-082-1554) can be seen easily in Figure 2d.

**Figure 4:**
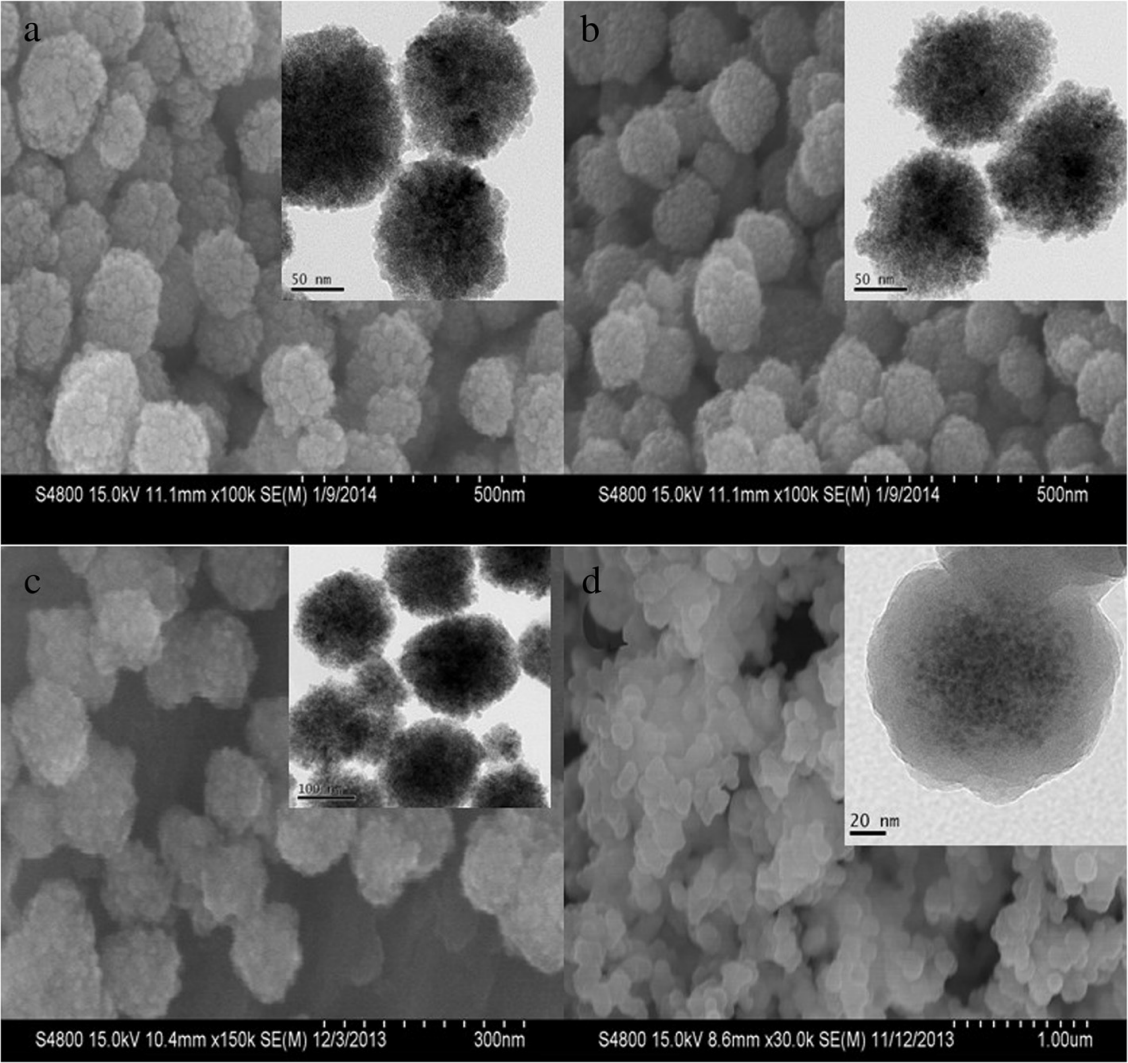
**SEM and TEM images of the synthesized: (a) naked Fe**_**3**_**O**_**4**_**, (b) CS-coated Fe**_**3**_**O**_**4**_**, (c) CdTe-CS-coated Fe**_**3**_**O**_**4**_**, and (d) Fe**_**3**_**O**_**4**_**-CdTe@SiO**_**2**_**-NH**_**2.**_

The SEM images in Figure 4a,b,c show that the as-prepared samples are composed of many nearly monodisperse spherical particles with a diameter of about 120 nm, the surfaces of these nanospheres are not smooth and are composed of many small nanoparticles, the possible driving force for such aggregation is the reduced surface energy of nanoparticles. However, the surface of CS-coated Fe_3_O_4_ NPs became smoother because of coating CS. In addition, comparing with the TEM images of naked Fe_3_O_4_ NPs (in Figure 4a inset), the CS-coated Fe_3_O_4_ NPs show that the mesoporous structure became more distinct (in Figure 4b inset). As shown in Figure 4c, comparing with CS-coated Fe_3_O_4_ NPs, after conjugation with CdTe QDs, the surface of CdTe-CS-coated Fe_3_O_4_ NPs became smoother. However, they were not significantly different from the TEM images of CS-coated Fe_3_O_4_ NPs because of small size of QDs [12].

**Figure 5:**
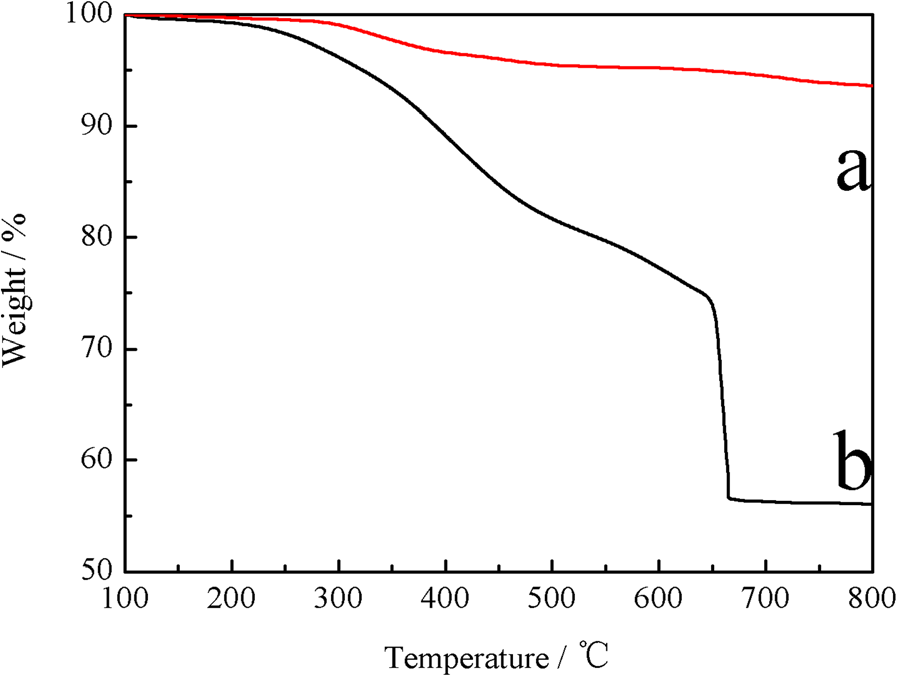
**TGA curves of (a) naked Fe**_**3**_**O**_**4 **_**NPs and (b) CS-coated Fe**_**3**_**O**_**4 **_**NPs.**

The TGA curves of naked Fe_3_O_4_, and CS-coated Fe_3_O_4_ NPs were shown in Figure 5. For naked Fe_3_O_4_, the TGA curve shows that the weight loss over the temperature range from 100 to 800°C was about 6.4%. This might be due to the loss of the removal of the remaining water and agents. Comparing with the TGA curves of the naked Fe_3_O_4_ NPs, the CS-coated Fe_3_O_4_ NPs show that the main mass of the as-synthesized occurred about 37.5% decreasing attributed to the decomposition of CS anchored on the surface of the Fe_3_O_4_ NPs.

**Figure 6:**
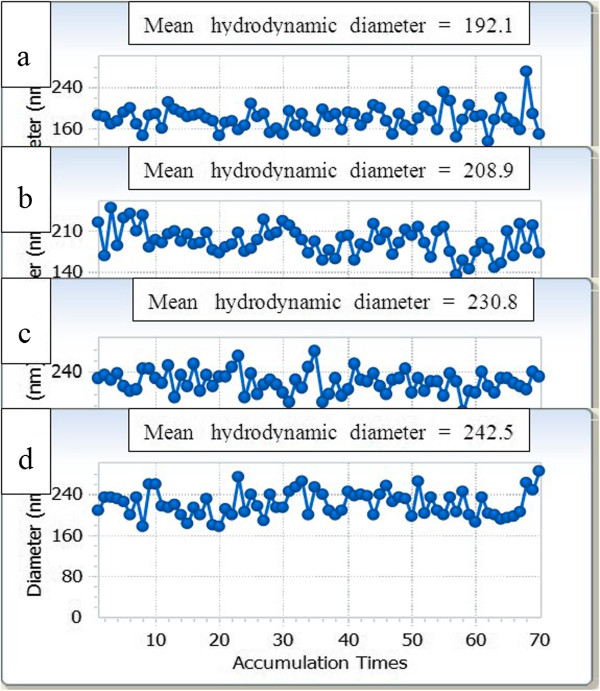
**The hydrodynamic diameter of as-prepared samples at pH =7.0: (a) naked Fe**_**3**_**O**_**4**_**, (b) CS-coated Fe**_**3**_**O**_**4**_**, (c) CdTe-CS-coated Fe**_**3**_**O**_**4**_**, and (d) Fe**_**3**_**O**_**4**_**-CdTe@SiO**_**2**_**-NH**_**2**_**.**

The hydrodynamic diameters of CS-coated Fe_3_O_4_ NPs (in Figure 6b), CdTe-CS-coated Fe_3_O_4_ NPs (Figure 6c) and Fe_3_O_4_-CdTe@SiO_2_-NH_2_ (Figure 6d) increased from 192.1 nm for naked Fe_3_O_4_ NPs (Figure 6a) to 208.9, 230.8, and 242.5 nm respectively. The result was likely caused by the CS, CdTe QDs, and SiO_2_ covered Fe_3_O_4_ NPs surface. As can be seen from these results, the particle size of as-synthesized NPs in the dry state was much smaller than that in aqueous medium because the diameters of NPs obtained by DLS reflected the hydrodynamic diameters of shell swollen in aqueous suspension, while those observed by SEM and TEM were the diameters of dried nanoparticles.

**Figure 7:**
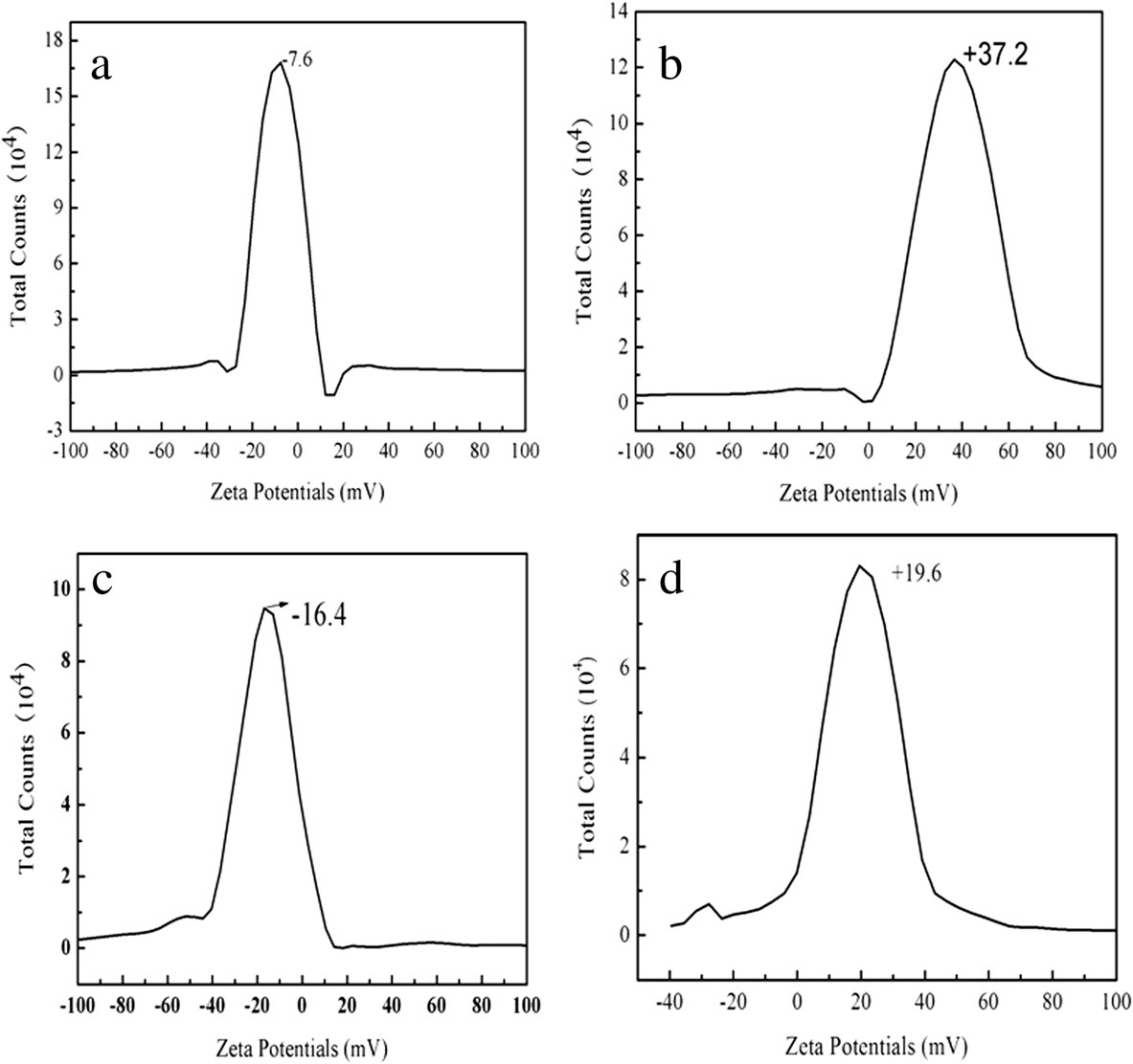
**The zeta potential of as-prepared samples at pH =7.0: (a) naked Fe**_**3**_**O**_**4 **_**NPs, (b) CS-coated Fe**_**3**_**O**_**4 **_**NPs, (c) CdTe-CS-coated Fe**_**3**_**O**_**4 **_**NPs, and (d) Fe**_**3**_**O**_**4**_**-CdTe@SiO**_**2**_**-NH**_**2 **_**NPs.**

The electrostatic interaction of the nanoparticles can be controlled by variation in their surface charges, which can be determined by measuring the zeta potential of these particles. Compared with naked Fe_3_O_4_ NPs (Figure 7a), the zeta potential of CS-coated Fe_3_O_4_ NPs possessed higher positive charge (Figure 7b). This may be caused by the protonation of the amino group (-NH_2_) in the chitosan. On the other hand, after coating CdTe QDs on the surface of CS-coated Fe_3_O_4_ NPs, the zeta-potential decreased from +37.2 to -16.4 as expected. Because CdTe QDs was conjugated to the amino group of CS, they neutralized the positive charge of CS. Besides, as-prepared CdTe QDs are strongly negatively charged moieties. As shown in Figure 7d, the zeta-potential was changed to about +19.6 after coated with SiO_2_-NH_2_ shell. This result indicates that CdTe-CS-coated Fe_3_O_4_ NPs could be successfully coated with SiO_2_-NH_2_ shell.

### Magnetic property and fluorescent spectra of the as-synthesized NPs

**Figure 8:**
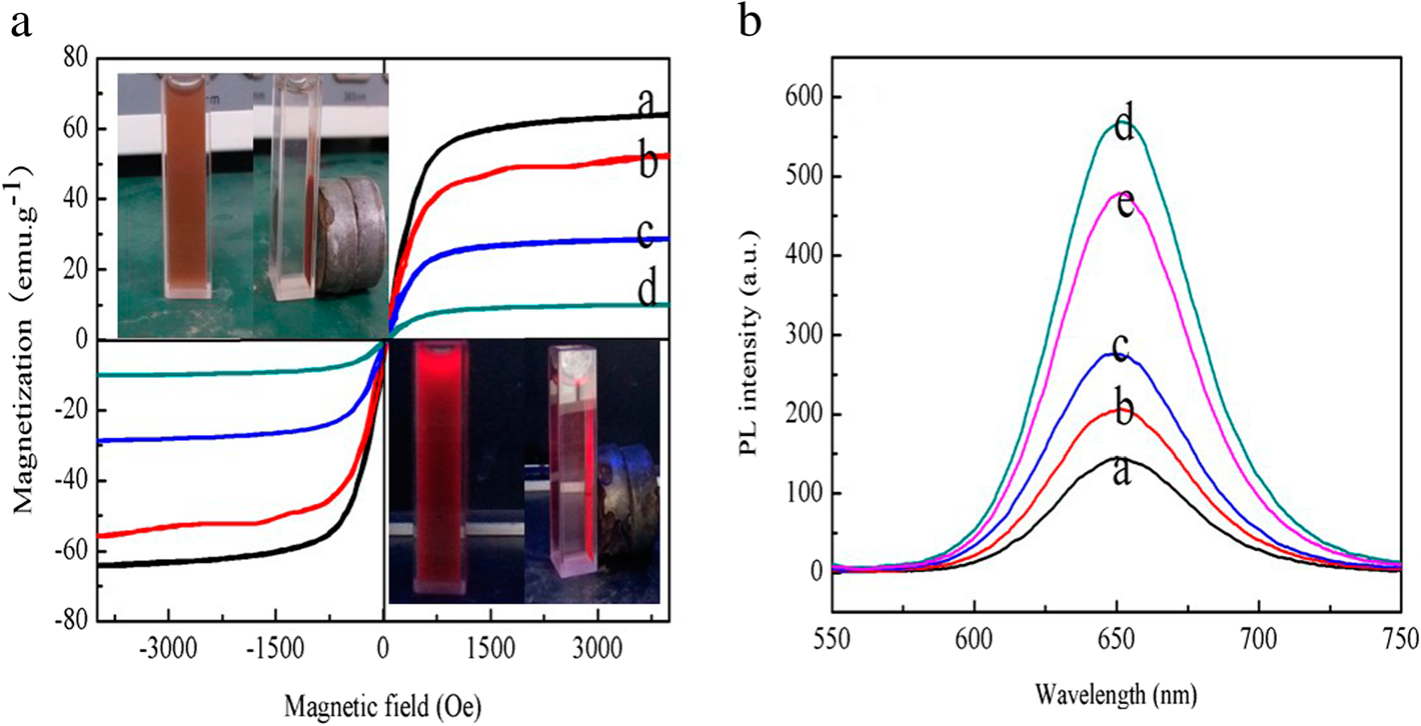
**Magnetization curves measured for the nanoparticles obtained. (a)** (a) naked Fe_3_O_4_ NPs, (b) CS-coated Fe_3_O_4_ NPs, (c) CdTe-CS-coated Fe_3_O_4_ NPs and (d) Fe_3_O_4_-CdTe@SiO_2_ NPs; The fluorescent spectra of the CdTe-CS-coated Fe3O4 NPs with different content of CdTe QDs. **(b)** (a) 10 ml CdTe QDs, (b) 20 ml CdTe QDs, (c) 30 ml CdTe QDs, (d) 50 ml CdTe QDs and (e) 60 ml CdTe QDs.

To gain a better understanding of the magnetic properties of as-synthesized NPs, the magnetization curves were produced. As shown in Figure 8a, neither coercivity nor remanence was observed, indicating the characteristic superparamagnetic behavior of nanocomposites, which is essential for magnetic separation. Moreover, Figure 8a (a) demonstrates that the saturation magnetization (Ms) of naked Fe_3_O_4_ NPs is 64.2 emu/g. The values of Ms of CS-coated Fe_3_O_4_ NPs, CdTe-CS-coated Fe_3_O_4_ NPs and Fe_3_O_4_-CdTe@SiO_2_-NH_2_ NPs were decreased to 52.0, 28.4, and 9.7 emu/g, successively. These phenomena can be explained by the diamagnetic contribution of the silica shell and the CdTe QDs surrounding the Fe_3_O_4_ magnetic core NPs.

Figure 8a (inset) illustrates the magnetic separation process of the Fe_3_O_4_-CdTe@SiO_2_ NPs under normal light (the upper left corner) and 365-nm excitation (the lower right corner). In the absence of an external magnetic field, the solution of Fe_3_O_4_-CdTe@SiO_2_-NH_2_ NPs is red and the NPs are well dispersed in the aqueous solution under both normal light and UV irradiation. When a magnetic field is placed near the solution, the as-prepared nanoparticles are attracted and accumulated toward the magnet, and the bulk solution becomes a clear phase, indicating that magnetic separation occurs. These results suggest that the as-prepared nanocomposites can find potential applications in magnetic guiding and separation. Figure 8b showed the fluorescent spectra of the CdTe-CS-coated Fe_3_O_4_ NPs with different content of CdTe QDs (Vol =10, 20, 30, 50, 60 ml) constructed by amide reaction. It is clearly seen that the photoluminescence (PL) intensity gradually increased first and then decreased with the content of CdTe QDs changing from 10 to 60 ml, which suggests the formation of more homogeneous QDs.

## Conclusion

In summary, a facile synthesis method was developed to prepare Fe_3_O_4_-CdTe@SiO_2_-FA NPs with tunable magnetism, sizes, suspension stability, and surface charge. The magnetic and fluorescent bifunctional microspheres have strong luminescent. Because these brightly luminescent beads exhibited high stability and super-paramagnetic behavior, we will next focus on their bio-applications, such as magnetic resonance imaging, drug delivery, cell labeling, and magnetic cell separation.

## Competing interests

The authors declare that they have no competing interests.

## Authors’ contributions

MS carried out the experiment and wrote the manuscript. WPJ participated in the data analysis. GC supervised the project. CPL, GDF, XYC, and YMJ provided the facilities and discussions related to them. All authors read and approved the final manuscript.
